# Fast-track surgery after gynaecological oncological surgery: study protocol for a prospective randomised controlled trial

**DOI:** 10.1186/s13063-016-1688-3

**Published:** 2016-12-15

**Authors:** Ling Cui, Yu Shi, GN Zhang

**Affiliations:** Department of Gynaecological Oncology, Sichuan Cancer Hospital, Chengdu, 610041 Sichuan People’s Republic of China

**Keywords:** Fast-track surgery, Gynaecological surgery, Oncological surgery, Post-operative length of hospitalisation, Randomised controlled study

## Abstract

**Background:**

Fast-track surgery (FTS), also known as enhanced recovery after surgery, is a multidisciplinary approach to accelerate recovery, reduce complications, minimise hospital stay without increasing readmission rates, and reduce health care costs, all without compromising patient safety. The advantages of FTS in abdominal surgery most likely extend to gynaecological surgery, but this is an assumption, as FTS in elective gynaecological surgery has not been well studied. No consensus guidelines have been developed for gynaecological oncological surgery although surgeons have attempted to introduce slightly modified FTS programmes for patients undergoing such surgery. To our knowledge, there are no published randomised controlled trials; however, some studies have shown that FTS in gynaecological oncological surgery leads to early hospital discharge with high levels of patient satisfaction. The aim of this study is whether FTS reduces the length of stay in hospital compared to traditional management. The secondary aim is whether FTS is associated with any increase in post-surgical complications compared to traditional management (for both open and laparoscopic surgery).

**Methods/design:**

This trial will prospectively compare FTS and traditional management protocols. The primary endpoint is the length of post-operative hospitalisation (days, mean ± standard deviation), defined as the number of days between the date of discharge and the date of surgery. The secondary endpoints are complications in both groups (FTS versus traditional protocol) occurring during the first 3 months post-operatively including infection (wound infection, lung infection, intraperitoneal infection), post-operative nausea and vomiting, ileus, post-operative haemorrhage, post-operative thrombosis, and the Acute Physiology and Chronic Health Enquiry II score.

**Discussion:**

The advantages of FTS most likely extend to gynaecology, although, to our knowledge, there are no randomised controlled trials. The aim of this study is to compare the post-operative length of hospitalisation after major gynaecological or gynaecological oncological surgery and to analyse patients’ post-operative complications. This trial may reveal whether FTS leads to early hospital discharge with few complications after gynaecological surgery.

**Trial registration number:**

NCT02687412. Approval Number: SCCHEC20160001. Date of registration: registered on 23 February 2016.

## Background

Fast-track surgery (FTS), also known as enhanced recovery after surgery, was initiated in 1995 by Bardram et al. [1]. FTS is a multidisciplinary approach to accelerate recovery, reduce complications, minimise hospital stay without increasing re-admission rates, and reduce healthcare costs, all without compromising patient safety [2]. FTS has been adopted by gynaecological, colorectal and upper GI specialities worldwide, and has been used successfully in non-malignant gynaecological surgery [3, 4], and is especially effective in elective colorectal surgery [5-7].

The speed of post-operative recovery is influenced by multiple factors including pain, post-operative nausea and vomiting (PONV), paralytic ileus, fatigue, and sleep disturbances. A multimodal approach to prevent and minimise these factors is considered essential to enhance recovery [2, 5, 6, 8]. Fast-track principles include providing the patient with thorough pre-operative information and education concerning pre-, intra- and post-operative care, the use of safe and short-acting anaesthetics, optimal dynamic pain relief with minimal use of opioids, management of PONV, enteral nutrition and early mobilisation, and the use of minimally invasive surgery [9].

The advantages of FTS documented in abdominal surgery most likely extend to gynaecological surgery; however, this is an assumption because FTS in elective gynaecological surgery has not been well studied. One study has shown that FTS in gynaecological oncology provides early hospital discharge and high levels of patient satisfaction [10]. However, no consensus guidelines have been developed for gynaecological oncological surgery although surgeons have attempted to introduce slightly modified FTS programmes for patients undergoing such surgery. To our knowledge, no randomised controlled trials have been published [3, 11, 12].

In traditional surgical care, patients are often admitted to hospital the day before the planned surgery, undergo pre-operative mechanical and antibiotic bowel preparation, and receive ongoing intravenous fluids to maintain fluid balance prior to surgery or anaesthesia. Intra-operatively, patients are often volume-loaded to maintain filling pressures, receive pelvic drains to prevent fluid collection, then spend 2–3 days nil by mouth until bowel sounds return before beginning a graduated diet of clear liquids, free fluids, light diet, and finally a regular diet 5–7 days post-surgery. Patients are discharged an average of 5–7 days post-surgery [13]. FTS or enhanced surgical recovery programmes have been developed and refined in many specialities with documented improved patient outcomes, earlier discharge from hospital, and reduced post-operative length of stay (LOS) [2, 14, 15].

The aim of this study is to analyse the post-surgical complications in patients receiving FTS who are discharged earlier than anticipated after major gynaecological or gynaecological oncological surgery.

## Methods/design

### Objectives and hypothesis

This prospective study will compare FTS and traditional management protocols and test the following hypotheses:

H0: length of stay and post-operative complications are equal in both groups.

H1: length of stay is enhanced in the FTS group and post-operative complications differ between groups.

### Study population and eligibility criteria

The trial is designed as a randomised, controlled, non-blinded, single-centre trial in the Department of Gynaecological Oncology of the Si Chuan Cancer Hospital Chengdu, Sichuan, China.

#### Inclusion criteria

1. Patients scheduled for gynaecological oncology surgery (including radical hysterectomy and lymphadenectomy, hysterectomy and lymphadenectomy, and cytoreductive procedures for both open and laparoscopic surgery);
2. Age: ≥ 18 years;
3. Signed informed consent provided.

#### Exclusion criteria

1. Patients with a documented infection at the time of surgery;
2. Age ≥ 71 years;
3. Patients with ileus at the time of surgery;
4. Patients with hypocoagulability;
5. Patients with psychological disorders, alcohol dependence, or drug abuse history;
6. Patients with primary nephrotic or hepatic disease;
7. Patients with severe hypertension defined as systolic blood pressure ≥ 160 mmHg and diastolic blood pressure > 90 mmHg.

#### Criteria for discontinuing

1. The trial appears to be causing unexpected harm or severe adverse events to participants, or evidence that the risks outweigh the benefits, with a discontinuance decision from the ethics committee.
2. The enrollment indicates the trial cannot be completed in the 3-month period.

### Sample size calculation

The sample size calculation is based on the LOS with a standard deviation in the traditional group of 1.5 based on previous studies [16, 17].

We estimate that in a superiority trial with an effect size of 90% and a margin of 10 (alpha 5%, power 90%) where μα = 1.96 and μβ = 1.28, using the equation n = [2(μα + μβ) σ /δ]^2^, a sample size of 47 patients per group is necessary to detect a difference between the groups. With an expected dropout rate of 20%, we plan to enrol 120 patients in the study.

### Method of generating the allocation sequence

Computer-generated random numbers and list of any factors for stratification. To reduce predictability of a random sequence, details of any planned restriction (e.g. blocking) should be provided in a separate document that is unavailable to those who enrol participants or assign interventions.

### Post-operative data collection

A daily assessment of the study patients will be made by clinical investigators or a delegated physician. Patients will be randomised when the surgery is booked. All protocol-required information collected during the trial will be entered into the patient’s record including: (1) patient characteristics, (2) hospitalisation information, (3) post-operative information, and (4) complications. Patient characteristics data will include: age, weight, height, body mass index, medical insurance status, blood pressure, glycaemia, lipidemia and performance status. Hospitalisation details will include LOS, the procedure performed, diagnosis, operating time, and intra-operative estimated blood loss. Post-operative details will include time to full tolerance of free fluids (days), time to full tolerance of solid food (days), and time to drain removal (days), admission to ICU, return to operating room, blood transfusion, venous thromboembolism (VTE), readmission to hospital, and malignancy status and hospitalisation expenses.

Complications (Table 1) details will include infection (wound infection, lung infection, intraperitoneal infection), PONV, ileus, post-operative haemorrhage, post-operative thrombosis, and Acute Physiology and Chronic Health Enquiry (APACHE) II score (Table 2).

**Table 1:** Checklist of fast track and traditional management

Allocation	FTS management	Traditional management
	Computer-generated random numbers	Computer-generated random numbers
Pre-operative		
	Pre-operative assessment, counselling and FT management education	No FT management education
	Information on the fast-track treatment and informed consent	Information on traditional treatment and informed consent
	Pre-operative nutritional drink up to 4 h prior to surgery (TPF-D produced by FreseniusKabi Deutschland GmbH, Bad Homburg, Germany). Fasting - solid food 6 h before and liquid food intake of clear fluids 2 h before anaesthesia	Pre-operative fasting at least 8 h
	Patients do not receive mechanical bowel preparation, only oral intestinal cleaner 12 h pre-operation can be accepted, but no need of liquid stool	Oral bowel preparation or mechanical bowel preparation until liquid stool
	Anti-microbial prophylaxis and skin preparation	Anti-microbial prophylaxis and skin preparation
	Pre-operative treatment with carbohydrates (10% glucose 400 ml orally 2–3 h before operation) (patients without diabetes)	No oral intake on the operation day
Intra-operative		
	Avoiding hypothermia, keeping the intra-operative core temperature at 36 ± 0.5 °C	Keeping the intra-operative core temperature at 34.7 ± 0.6 °C
	Anti-emetics at end of anaesthesia	Not every patient gets anti-emetics at end of anaesthesia
Post-operative		
	Post-operative glycaemic control	Post-operative glycaemic control only with diabetes
	Preventive post-operative nausea and vomiting (PONV) control	Post-operative nausea and vomiting (PONV) control when it happens
	Early post-operative diet (3–6 h after surgery, patients resume a liquid diet, 12 h after surgery patients begin to take solid diet)	6 h after surgery, patients resume a liquid diet, patients begin to take solid diet after anal exhaust
	Early mobilisation	Early mobilisation
	Time to drain removal less than 24 h (eliminate post-operative bleeding and urinary fistula, intestinal fistula)	Time to drain removal less than 48 h (eliminate post-operative bleeding and urinary fistula, intestinal fistula)
Audit	Systematic audit improves compliance and clinical outcomes

**Table 2:** Clinical parameters and post-operative complications for analysis

Parameters	Definitions
Patient characteristics	Age, weight, height, body mass index (BMI), medical insurance status and performance status
Hospitalisation	LOS (length of hospitalisation post-operation), the procedure performed, diagnosis, operating time, name of surgery, intra-operative estimated blood loss
Post-operation	Time to full tolerance of free fluids (days), time to full tolerance of solid food (days), time to drain removal (days) hospitalisation expenses
Complications	
Infection	Wound infection, lung infection, intraperitoneal infection, operation space infection (fever, mild abdominal pain without radiographic abnormalities)
Post-operative nausea and vomiting (PONV)	It was recognised that nausea and vomiting are common side effects of surgical recovery
Ileus	Is a disruption of the normal propulsive ability of the gastrointestinal tract
Post-operative haemorrhage	Evidence of blood loss from drains or based on ultrasonography
Post-operative thrombosis	Evidence of blood thrombosis based on ultrasonography
APACHE II score	Acute Physiology and Chronic Health Evaluation II

### Primary and secondary endpoints

#### Primary endpoints

LOS (days (d), mean ± standard deviation (SD)), which is defined as the number of days between the date of discharge and the date of surgery.

#### Secondary endpoints

Complications: complications in both groups are assessed during the first 21 days hospitalisation expenses post-operatively and include infection (wound infection, lung infection, intraperitoneal infection), PONV, ileus, post-operative haemorrhage, post-operative thrombosis, and APACHE II score (Table 2).

### Ethics, study registration and consent

This trial was approved by an independent ethics committee at Sichuan Cancer Hospital and Research Institute.

Board Affiliation: Sichuan CHRI

Telephone: +86 02885420681; email: scchgcp@163.com

The study procedures, risks, benefits and data management will be discussed with patients before they are asked to provide informed consent to participate.

### Study treatment

**Fig. 1:**
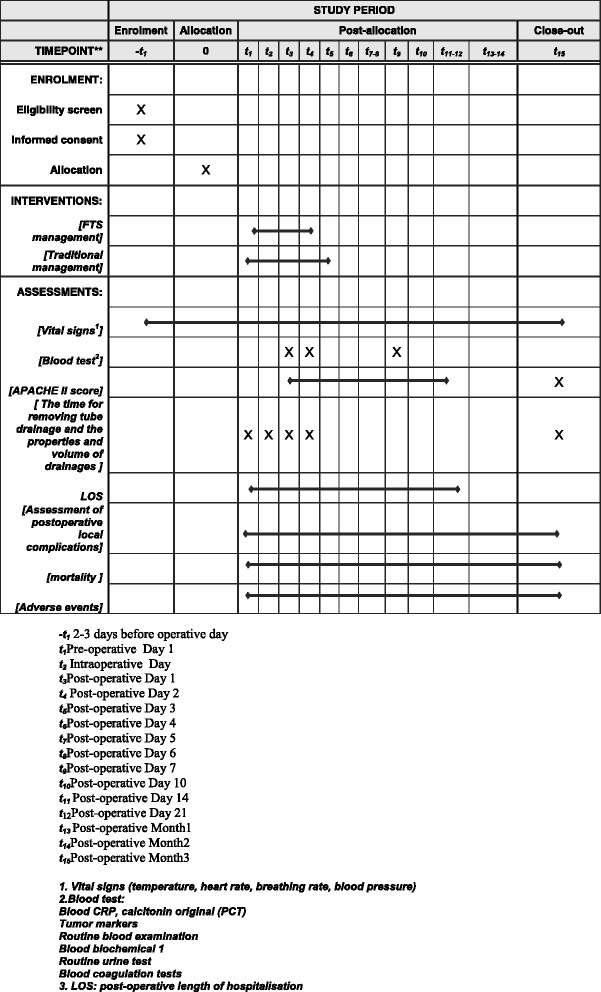
Content for the schedule of enrollment, intervention, and assessments

The surgical technique is standardised for the treatment team, and patients’ families are not blinded to the study. Data collectors are not involved in the clinical management of patients to ensure statistical validity and reliability. All surgeries are performed by the same team of surgeons, and patients are treated and nursed by the same treatment team during the pre-operative period. Post-operative complications are based on patient complaints and clinical symptoms. Given that there are no FTS guidelines for gynaecological oncological surgery, we refer to the guidelines for gastrectomy, colorectal surgery, and pancreaticoduodenectomy (Table 2 and Fig. 1) [18–20].

### Safety

Gynaecological oncological surgery is highly technically demanding. To avoid bias based on the surgical learning curve, every surgical procedure will be performed or supervised by a senior surgeon. Informed consent will be obtained from all participants.

### Methods for avoiding bias

#### Minimising systemic bias

**Fig. 2:**
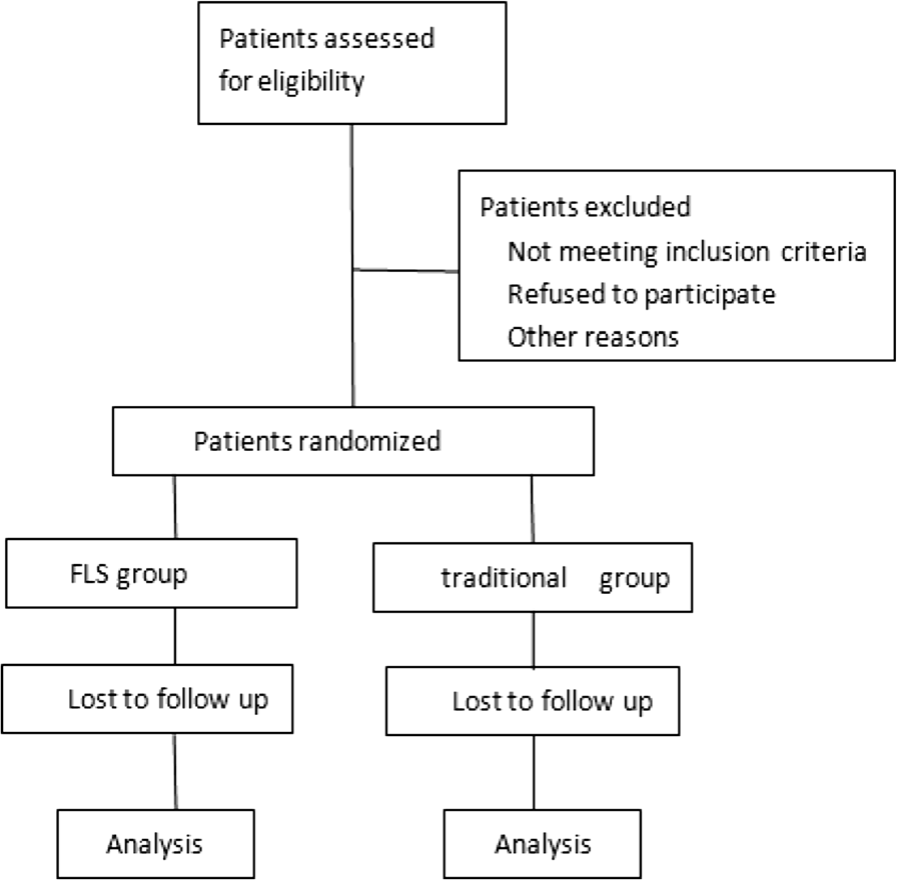
Study flow diagram

Patients will be randomised to one of the two groups after admission. Randomisation will be accomplished using balanced permutation blocks by generating random numbers to obtain homogeneity between groups. Opaque, sealed envelopes will be labelled with the randomisation number and will contain a sheet stating the group allocation for the patient. Randomisation envelopes will be used in consecutive order. Basic patient characteristics and the day of randomisation will be documented on a data sheet so that compliance to the randomisation scheme can be checked retrospectively. If patients are excluded from the study after randomisation, their numbers will not be reused. Operating surgeons, attending physicians, nursing staff, and patients and families cannot be blinded in this study, as the procedures differ between groups; however, outcome assessors will not be blinded. The randomisation process will follow the CONSORT guidelines (Fig. 2) [21].

#### Minimising treatment bias

Gynaecological oncological surgery (including radical hysterectomy and lymphadenectomy, hysterectomy and lymphadenectomy, and cytoreductive procedures) are standardised in both groups, and all surgeons participating in the study are familiar with these procedures, which are commonly and routinely performed, thus eliminating a learning curve.

#### Minimising measurement bias

LOS and post-operative complications, which are the primary and secondary endpoints, will be based on data in the patient’s record. Blinding is not necessary, because the LOS is an objective endpoint that cannot be influenced by the patient. Physician blinding is not possible because they perform the surgery.

### Patient pathway

All the patients in the trial will go through as depicted in the flowchart (Fig. 2).

### Statistical methods

Each patient’s allocation to the analysed population will be defined prior to the analysis and will be documented. In the full analysis set, patients will be analysed as randomised according to the intention-to-treat principle. The intention-to-treat principle implies that the analysis includes all randomised patients. The per protocol analysis set will include all patients without major protocol deviation. Deviations from the protocol will be assessed as major or minor, and patients with major deviations from the protocol will be excluded from the per protocol analysis. The safety analysis set will analyse patients according to the treatment.

The null hypothesis assumes that LOS and post-operative complications are equal in both groups. A binary logistic regression will be used to compare LOS between groups while adjusting for other factors.

Data will be analysed using SPSS 19.0 (IBM Corp., Armonk, NY, USA) and expressed as mean ± SD. LOS in the FTS and traditional groups will be compared and analysed using the Mann-Whitney *U* test (non-normal distribution). NRS2002 scores between the two groups will be analysed using Wilcoxon’s test (non-normal distribution) or Student’s *t* test (normal distribution). The chi-square test or Fisher’s exact test will be used to analyse the categorical secondary endpoints (complications). *P* < 0.05 will be considered statistically significant.

## Discussion

FTS has been adopted by most surgical specialties worldwide; however, few studies have assessed FTS in gynaecological malignant surgery [22], and there are currently no randomised controlled trials to support or refute this approach [11]. To our knowledge, no consensus guidelines have been developed for gynaecological oncological surgery although surgeons have attempted to introduce slightly modified FTS programmes for patients undergoing such surgery [3, 11, 12].

Widespread education is needed to improve the rate of implementation of FTS. There are several possible reasons for the lack of implementation including a lack of collaboration within surgical teams and a lack of awareness of or failure to accept and adopt evidence-based findings [8, 9, 23]. Close cooperation between the surgical, anaesthestic, and nursing staff is essential, and the importance of cooperation cannot be overestimated as practice using FTS is needed to achieve further developments in surgical care and post-operative recovery [24, 25]. Fast-track regimens have been well evaluated, generally, regarding medical complications, and they appear to be safe [26].

The aim of this study is to compare LOS and to analyse post-operative complications after major gynaecological or gynaecological oncological surgery. This trial may reveal whether FTS results in early hospital discharge and low complication rates after gynaecological surgery.

### Trial status

At the time of writing, we are about to enrol patients, and the anticipated study completion date is May 2017.

## Abbreviations

APACHE II score: Acute Physiology and Chronic Health Evaluation II
FTS: fast-track surgery
LOS: postoperative length of hospitalisation
PONV: postoperative nausea and vomiting
